# Feasibility study for dose calculation with a radiation treatment planning system using a fixed-size electron cone applicator for small electron fields

**DOI:** 10.1371/journal.pone.0324722

**Published:** 2025-08-14

**Authors:** Su Chul Han, Min Cheol Han, Jihun Kim, Heerim Nam, Jin Sung Kim, Kwangwoo Park

**Affiliations:** 1 Department of Radiation Oncology, Kangbuk Samsung Hospital, Sungkyunkwan University School of Medicine, Seoul, Republic of Korea; 2 Department of Radiation Oncology, Yonsei University College of Medicine, Seoul, Republic of Korea; Northwestern University Feinberg School of Medicine, UNITED STATES OF AMERICA

## Abstract

**Objective:**

This study aims to evaluate the feasibility of radiation treatment planning using a commercial treatment planning system (TPS) for small fixed-size electron cone electron applicators not natively supported by the TPS.

**Methods:**

Dosimetric characteristics, including beam profiles and output factors (OFs), were compared between a 6 MeV electron beam collimated by a small fixed-size electron cone applicator and a cerrobend cutout-based general applicator. Measurements were performed using a micro-diamond detector in a water phantom for field sizes of 2, 3, 4, and 5 cm. The monitor units (MUs) from the TPS were compared with direct measurements. To estimate the MU for the fixed-size electron cone applicator using the TPS, the relative OFs were defined as the ratio of the OFs for the fixed-size electron cone and general applicators. Dose distributions obtained from the TPS were validated against measurements using Gafchromic films, ensuring accuracy.

**Results:**

Gamma analysis showed a passing rate >95% with 1%/1 mm criteria for depth dose comparisons and >99% with 2%/2 mm criteria for beam profiles. The general applicator’s OFs were consistently higher across all measured field sizes. The MU difference between the TPS and measurements was within 2.0%, while the difference between indirect TPS calculations and direct measurements for the fixed-size electron cone applicator remained within 1.0%. Dose distribution analysis showed >99% agreement (3%/3 mm) between the 2D dose distribution obtained using film in the fixed-size electron cone applicator and that calculated by the TPS of cerrobend cutout-based applicator.

**Conclusion:**

The results demonstrate the feasibility of calculating monitor units and dose distributions for small fixed-size electron cone applicators using a commercial TPS combined with relative output factors. This approach offers a reliable method for dose calculation in specialized electron therapy applications.

## Introduction

Electron beam radiotherapy has advantages over photon beam radiotherapy in treating cancers near the skin due to more effective sharp-dose falloff with depth [1]. Electron beam therapy requires the use of specialized applicators, which may vary depending on the manufacturer and treatment intent. Although these applicators are primarily designed to collimate the beam and minimize scatter near the patient’s surface, structural differences between applicator types can result in measurable dosimetric variations. In intraoperative radiation therapy (IORT), for example, the applicator design differs significantly from those used in conventional electron beam radiotherapy [2–4]. Compared to the specific photon beam, it has a characteristic that the dose drops steeply with depth, which is advantageous for treating cancer near the skin For new radiotherapy equipment, medical physicists typically measure beam data and input it into a treatment planning system (TPS) to ensure accurate dose calculations. Standard electron beam applicators, often used with Cerrobend cutouts, allow for precise beam shaping, and their dosimetric properties are well-established within TPS environments [5]. However, for specialized cases such as skin cancer treatment or keloid irradiation, certain manufacturers provide fixed-size electron cone applicators, which are stainless steel structures designed to generate small circular fields (2, 3, 4, and 5 cm in diameter). These applicators are not directly supported by commercial TPS platforms, requiring medical physicists to manually determine monitor units (MUs) based on empirical measurements—a process that is both time-consuming and prone to uncertainty.

Previous studies, such as that by Venanzio et al. [6] have compared the dosimetric characteristics of cerrobend cutout-based applicators and fixed-size electron cone applicators, demonstrating similar beam profile behavior. However, their study did not quantitatively analyze how these applicators can be integrated into a TPS for monitor unit calculations and dose distribution evaluation. To address this gap, our study investigates the feasibility of calculating MUs and dose distributions for fixed-size electron cone applicators using a commercial TPS.

This study proposes an indirect calculation method, using relative output factors (OFs), to estimate the MU required for fixed-size electron cone applicators. We compare TPS-calculated dose distributions with experimental measurements to assess whether this method can provide clinically acceptable accuracy. By introducing a systematic approach for integrating fixed-size electron cone applicators into TPS workflows, this study aims to enhance the efficiency and reliability of treatment planning for small-field electron beam radiotherapy.

## Materials and methods

### Electron applicator for radiotherapy and overall concept of this study

**Fig 1:**
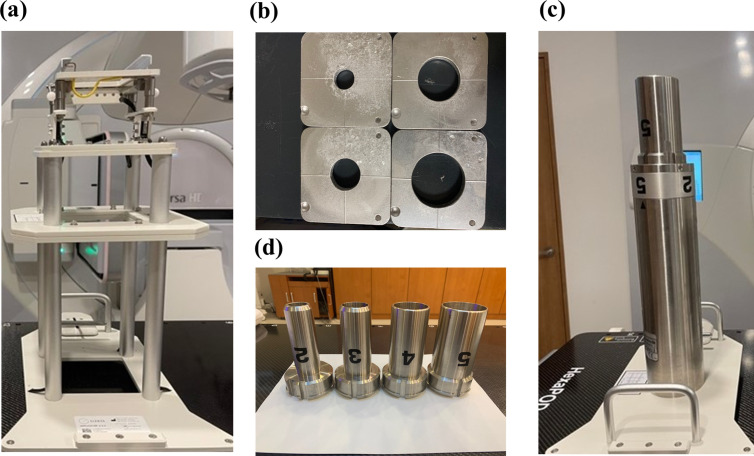
Two applicators in electron radiotherapy. (a) 6 cm × 6 cm electron applicator; (b) circular cerrobend cutout (c) fixed-size electron cone applicator (d) removable stainless end tube.

An applicator provided by the equipment manufacturer (Elekta, Sweden) is shown in Fig 1a; a custom-made cerrobend cutout (Fig 1b) with different diameters corresponding to the patient’s tumor size can be installed at the end of the applicator. The equipment manufacturer provided applicators with diameters of 6, 10, 14, 20, and 25 cm. Fig 1c shows a special electron applicator (fixed-size electron cone applicator) for irradiation in small circular fields 2, 3, 4, and 5 cm in diameter from the bottom tip of the stainless-steel applicator. As shown in Fig 1d, it can be freely replaced according to the field size. The cylindrical shape enclosed the electron beam path of the special electron applicator and was equal to the geometric height of the 6 cm × 6 cm electron applicator provided by the manufacturer. Additional data comparing applicator configurations are available in S1 File (S1 Data)

**Fig 2:**
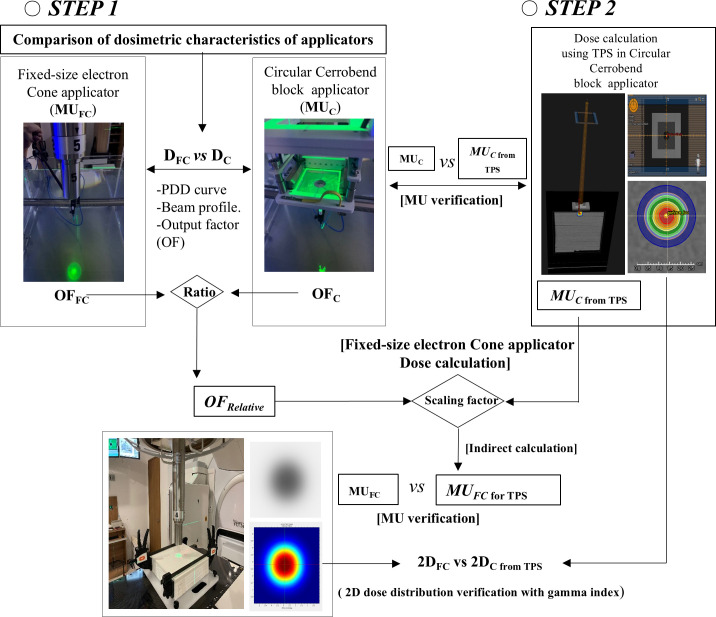
Overall concept for feasibility study of dose calculation with TPS in fixed-size electron cone applicator.

To evaluate the possibility of dose calculation with the TPS for a fixed-size electron cone applicator with a 6 MeV electron beam, this study was conducted in two steps (Fig 2).

In step one, to compare the dosimetric characteristics of the applicators ($D_{c}$,$\;D_{FC})$, we measured the beam quality, beam profile, and output factor of the cerrobend cutout-based general applicator and the fixed-size electron cone electron applicator.

In step two, the irradiation conditions of the circular cerrobend cutout-based general applicator were modeled using TPS (RayStation ver. 5.0), and the 2D dose distribution calculated using the TPS was compared with that measured using a gafchromic film (EBT3, Gafchromic Film, Ashland, Inc.) in a fixed-size electron cone applicator. The possibility of dose calculation was evaluated without direct modeling of the fixed-size electron cone applicator in the TPS. As shown in Fig 2, a method was suggested to calculate the MU (${MU}_{FC\;for\;TPS}$) of the fixed-size electron cone applicator for the TPS using the MU (${MU}_{c\;from\;TPS}$) calculated from the TPS and the relative OF (${OF}_{relative}$) for both applicators as scaling factor. The MU (${MU}_{FC\;for\;TPS}$) calculated indirectly and the ${MU}_{FC}$ based on direct measurements in the fixed-size electron cone applicator were compared and analyzed.

### Comparison of dosimetric characteristics of applicators at 6 MeV

In step one, beam data were obtained at 6 MeV using a Beamscan 3D water phantom (PTW, Freiburg, Germany) and a microdiamond detector (PTW 60019, Freiburg, Germany) to compare the dosimetric properties of the applicators. The gamma analysis was evaluated using Mephysto Data analyze 4.3 (PTW, Freiburg, Germany), designated as ref. (cerrobend cutout-based general applicator) and target (fixed-size electron cone applicator).

Electron beam quality is generally defined as $R_{50}$, and calculated from the PDD curve. The difference in the PDDs between the applicators was evaluated based on the field size and SSD. To assess SSD-dependent dosimetric variations, PDD and output factor measurements were conducted at three SSDs (95 cm, 100 cm, and 105 cm), whereas beam profiles were measured at a fixed SSD of 100 cm.

Beam profile measurements were performed in two directions (cross- and in-plane) at five depths, $R_{100}$, $R_{90}$, $R_{70}$, $R_{50}$, and1/2 $R_{90}$, measured from the PDD curves according to the field size of the applicators. The field sizes were measured at 50% of the central axis dose value; 80–20% penumbra values were measured in $R_{100}$. The differences in PDD curves and beam profiles between the applicators were analyzed using a gamma index (2%/ 2 mm).

The output factor (OF, $S_{e}$) was calculated using equation 1 defined in TG-70 as the ratio of the dose at $R_{100}$ of the small field ($r_{a}$, 2 cm, 3 cm, 4 cm, 5 cm) and the reference field ($r_{0}$, 10 cm × 10 cm) for the same MU. [7]

The OFs measured in the circular cerrobend-cutout applicator were defined as ${OF}_{c}$; those measured in the fixed-size electron cone applicator were defined as ${OF}_{FC}$. Equations 2 were used to analyze the differences in OFs according to the type of applicator.


$$\text{Output\textbackslash{} factor }{=S}_{e}\left(r_{a},\;SSD\right)=\;\frac{D/MU(d_{m}\left(r_{a}\right),r_{a},\;SSD)}{D/MU(d_{m}\left(r_{0}\right),r_{0},\;{SSD}_{0})}$$ (1)

$$\text{Diff. }(\;\%)\;=\frac{{OF}_{C}-{OF}_{FC}}{{OF}_{FC}}\;\times 100$$ (2)

Additional raw data used in this analysis are provided in S2 Table (S1 Data)

### Comparison of dose distribution for applicators with gafchromic film

**Fig 3:**
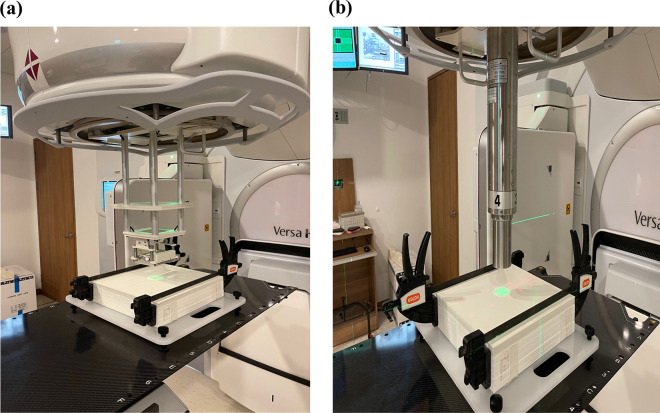
Comparison of dose distributions for both applicators with gafchromic film: (a) circular cerrobend-cutout applicator; (b) fixed-size electron cone applicator.

A gafchromic film was used to compare and analyze the 2D dose distributions in both applicators. As shown in Fig 3, the gafchromic film was located at the same position (Depth: 14 mm) in the slab phantom for both applicators; 500 MU was delivered to the film to provide sufficient darkness.

To analyze the dose distribution in both applicators, a calibration curve between the known dose and the optical density of the film was obtained from 6 MV prior to the film being from the same lot as that of the film used in this study. The film dosimetry process was performed according to the AAPM TG 235 guideline [8]. The dose distribution obtained from each applicator was normalized based on the maximum dose. The difference in dose distribution of the applicators was evaluated using gamma evaluation (3%/3 mm). All film dosimetry processes were performed using the RIT 113 film dosimetry system software (RIT Inc., Denver, Colorado).

### Comparison of MU and dose distribution between applicators and TPS

In step two, the irradiation conditions were the same as those used for the cerrobend cutout-based general applicator in the TPS; the diameters of the circular cutout were 2, 3, 4 and 5 cm at the end of the applicator. The dose calculation of the TPS was performed using the Monte Carlo V.3.2) algorithm of the RayStation TPS with a dose grid of 2 mm and 1,000,000 particles per unit area (cm^3^). To calculate ${MU}_{c\;from\;TPS}$ in the TPS, a dose of 500 cGy was designed to be irradiated to a specific depth (1.4 cm) in the ${SSD}_{0}(100\;cm)$, depending on the small field size ($r_{a}$: 2, 3, 4 and 5 cm). The MU based on the measurements was calculated for both applicators according to TG 71 [9] (equation 3). The calculated MUs were defined as ${MU}_{C}$ (circular cerrobend-cutout applicator) and ${MU}_{FC}$ (fixed-size electron cone applicator).


$$\text{MU }=\;\frac{D\cdot 100\%}{D_{0}^{\prime }\;\cdot PDD\left(d,\;r_{a},\;{\;SSD}_{0}\right)\;\cdot \;S_{e}(r_{a},\;{SSD}_{0})}$$ (3)

The ${MU}_{c\;from\;TPS}$ was compared with the ${MU}_{c\;}$ for the circular cerrobend-cutout applicator according to the field size. To calculate ${MU}_{FC\;for\;TPS}$ for a fixed-size electron cone applicator in the TPS, ${MU}_{c\;from\;TPS}$ was calculated from the TPS and the relative OF $\left({OF}_{relative}\right)$, as in equation 4. ${OF}_{relative}$ is defined in equation 5 as the OF ratio of each applicator for the same field size. The calculated ${MU}_{\;FC\;for\;TPS}$ was compared with that of the ${MU}_{FC\;}$, according to the field size.


$${MU}_{\;FC\;for\;TPS\;}=\;\frac{{MU}_{\;C\;from\;TPS\;}}{{OF}_{relative}}$$ (4)

$${OF}_{relative}=\frac{{OF}_{Fc}\;(r_{a},\;SSD)}{{OF}_{c}\;(r_{a},\;SSD)}$$ (5)

The dose distribution calculated from the TPS for the circular cerrobend-cutout applicator was compared to that measured using a film in a fixed-size electron cone applicator. The difference in dose distribution was evaluated using gamma evaluation (3%/ 3 mm). All film dosimetry processes were performed using the RIT 113 film dosimetry system software.

## Results

### Beam quality depending on field size and SSD for both applicators

Table 1 shows the beam quality of 6 MeV electrons measured in both applicators across different field sizes and SSDs. In smaller fields (e.g., 2–3 cm), slight differences were observed in R₁₀₀ and R₅₀ between the two applicators, with the fixed-sized electron cone applicator showing marginally greater penetration. However, for larger fields (≥4 cm), the values for R₁₀₀, R₅₀, and R_p_ were nearly identical between the applicators and consistent with the reference 10 × 10 cm field, indicating that beam quality differences were negligible at clinical field sizes.

**Table 1:** Comparison of beam quality of applicators depending on field size and SSD (95, 100, and 105 cm).

Applicator type		Circular cerrobend-cutout + 6 cm applicator (C)				Fixed-size electron cone applicator (FC)				Ref. filed (10 cm × 10 cm)
Field size		2 cm	3 cm	4 cm	5 cm	2 cm	3 cm	4 cm	5 cm	
$R_{100\;}$ (mm)	95 cm	10.00	13.01	14.07	14.01	9.02	13.02	14.0	14.00	13.98
	100 cm	9.01	12.99	14.00	14.00	9.99	13.01	14.01	14.00	14.00
	105 cm	9.01	12.99	14.00	14.00	9.99	13.01	14.01	14.00	13.97
Average ± S.D (mm)		9.34 ± 0.57	13.00 ± 0.01	14.02 ± 0.04	14.00 ± 0.00	9.67 ± 0.56	13.01 ± 0.01	14.00 ± 0.01	14.00 ± 0.00	13.98 ± 0.01
$R_{50}$ (mm)	95 cm	23.01	25.21	25.48	25.66	23.27	25.16	25.68	25.44	25.16
	100 cm	23.19	24.93	25.30	25.34	23.74	24.97	25.25	25.31	25.06
	105 cm	23.19	24.93	25.30	25.34	23.74	24.97	25.25	25.31	25.14
Average ± S.D (mm)		23.13 ±0.10	25.02 ±0.16	25.36 ±0.10	25.44 ± 0.18	23.58 ±0.27	25.03 ±0.10	25.39 ±0.24	25.35 ± 0.07	25.12 ± 0.04
$R_{p\;}$ (mm)	95 cm	31.62	32.10	31.86	32.07	31.58	31.09	32.04	31.91	31.52
	100 cm	31.94	31.72	31.70	31.72	31.93	31.82	31.72	31.68	31.47
	105 cm	31.94	31.72	31.70	31.72	31.93	31.82	31.72	31.68	31.54
Average ± S.D (mm)		31.83 ±0.18	31.84 ±0.21	31.75 ±0.09	31.83 ± 0.20	31.81 ±0.20	31.57 ±0.42	31.82 ±0.18	31.75 ± 0.13	31.51 ± 0.03

**Fig 4:**
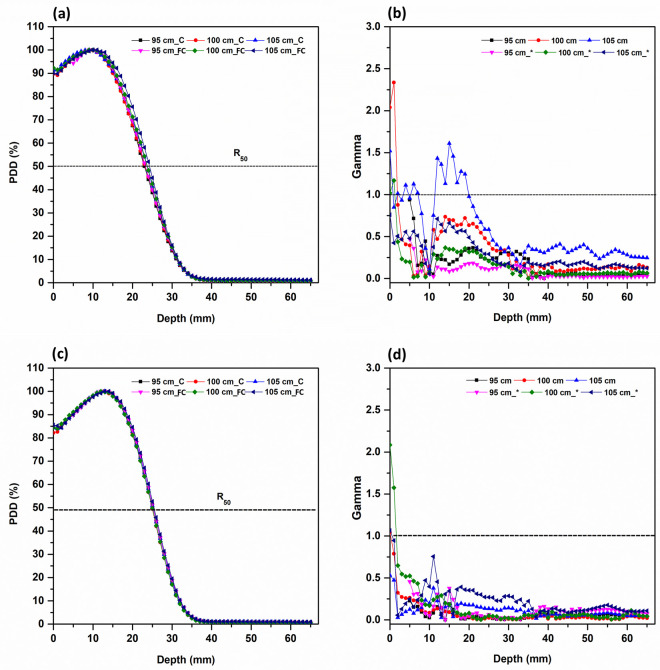
PDD curves for 2, 3 cm diameter field and gamma index comparison between the circular cerrobend-cutout applicator and the fixed-size electron cone applicator at different SSDs. (a) PDD curves of 2 cm, (b) Gamma index (2%/ 2 mm) of 2 cm, (c) PDD curves of 3 cm, (d) Gamma index (2%/ 2 mm) of 3 cm with * indicating 1%/ 1 mm. SSD_C refers to the measurement SSD in the circular cerrobend-cutout applicator, and SSD_FC refers to the measurement SSD in the fixed-size electron cone applicator. The dotted line represents a gamma index of 1.0.

**Fig 5:**
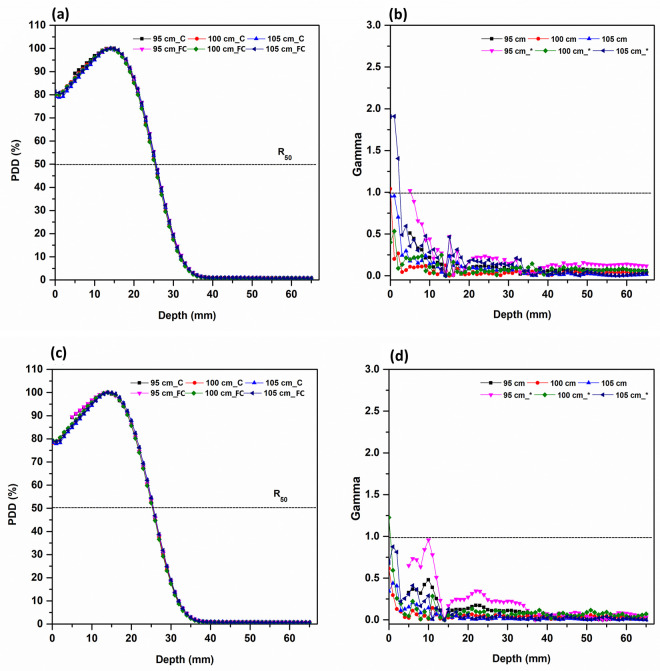
PDD curves for 4, 5 cm diameter field and gamma index comparison between the circular cerrobend-cutout applicator and the fixed-size electron cone applicator at different SSDs. (a) PDD curves of 4 cm, (b) Gamma index (2%/ 2 mm) of 4 cm, (c) PDD curves of 5 cm, (d) Gamma index (2%/ 2 mm) of 5 cm with * indicating 1%/ 1 mm. SSD_C refers to the measurement SSD in the circular cerrobend-cutout applicator, and SSD_FC refers to the measurement SSD in the fixed-size electron cone applicator. The dotted line represents a gamma index of 1.0.

For a 2 cm field, when the gamma index was set to 2%/ 2 mm, the passing rate exceeded 95% for all SSD except at certain surface depth; when the gamma index was set to 1 mm/ 1%, the result was over 95% for SSD 95 cm and SSD 100 cm; for SSD 105 cm, it was 81.2%(Fig 4). In 3, 4, 5 cm field, the evaluation results shows that PDD consistency for all but the depth of some surfaces was 95% or more and passed at 2%/ 2 mm; 1%/ 1 mm showed similar (95% or greater) pass rates(Fig 5).

### Beam profile depending on field size and depth for both applicators

Table 2 summarizes the characteristics of the beam profiles measured using both applicators. The measurement depths were $R_{100}$, $R_{90}$, $R_{70}$, $R_{50}$, and1/2 $R_{90}$, as measured from the PDD curves with an SSD of 100 cm for each field size. The FWHM and both penumbras were compared in $R_{100}$ as beam characteristics. The FWHM of the circular cerrobend-cutout applicator was smaller than that of the fixed-size electron cone applicator. In the penumbra, the result for the circular ceramic cutout-based applicator was smaller than that for the fixed-size electron cone applicator; the average difference was within 1 mm.

**Table 2:** Comparison of beam profiles of applicators depending on field size and depth (SSD = 100 cm, FWHM and both penumbras were evaluated in $R_{100\;}$).

Applicator type		Circular cerrobend-cutout + 6 cm applicator (C)				Fixed-size electron cone applicator (FC)				Diff. (C -FC) (average ± S.D. mm)
Field size		2 cm	3 cm	4 cm	5 cm	2 cm	3 cm	4 cm	5 cm	
Depth (mm)	$R_{100}$	9.01	12.99	14.0	14.0	9.99	13.01	14.01	14.0	0.25 ± 0.42
	$R_{90}$	15.33	18.38	19.16	19.35	15.53	18.36	19.27	19.11	−0.01 ± 0.17
	$R_{70}$	19.50	23.30	22.90	21.6	20.00	23.30	23.00	23.00	0.50 ± 0.56
	$R_{50}$	23.19	24.93	25.30	25.34	23.74	24.97	25.25	25.31	−0.13 ± 0.25
	1/2$R_{90}$	7.67	9.19	9.58	9.68	7.77	9.18	9.64	9.56	−0.00 ± 0.08
FWHM (mm)	In- plane	20.80	31.30	41.70	53.20	22.70	32.50	42.90	53.60	−1.2 ± 0.50
	Cross-plane	20.90	31.00	41.60	53.10	22.60	32.50	43.00	53.60	−1.3 ± 0.40
Lt, Pen. (mm)	In- plane	7.26	9.31	10.49	10.78	7.49	9.70	11.07	11.30	−0.43 ± 0.13
	Cross-plane	7.16	9.35	10.48	10.74	7.43	9.54	11.09	11.74	−0.51 ± 0.40
Rt, Pen. (mm)	In- plane	7.40	9.48	10.44	10.61	7.50	9.60	10.88	11.33	−0.34 ± 0.25
	Cross-plane	7.47	9.57	10.53	10.73	7.47	9.35	10.72	11.61	−0.21 ± 0.41

**Fig 6:**
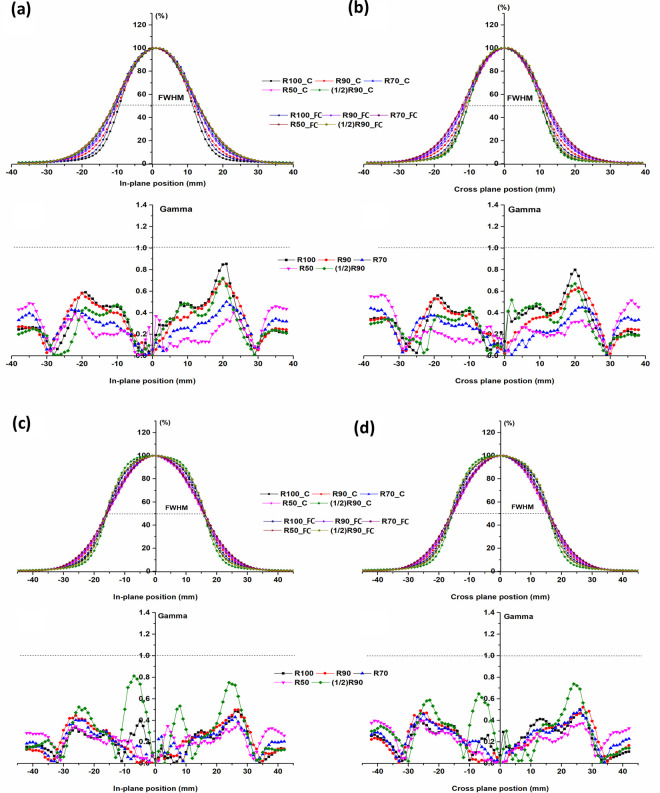
Plane-normalized profiles for 2, 3 cm diameter field and gamma index comparison (2%/ 2 mm) for applicators, at different depths. (a) In-plane of 2 cm, (b) Cross-plane of 2 cm, (c) In-plane of 3 cm, (d) Cross-plane of 3 cm (SSD: 100 cm. The dotted line represents a gamma index of 1.0 at 2%/2 mm).

**Fig 7:**
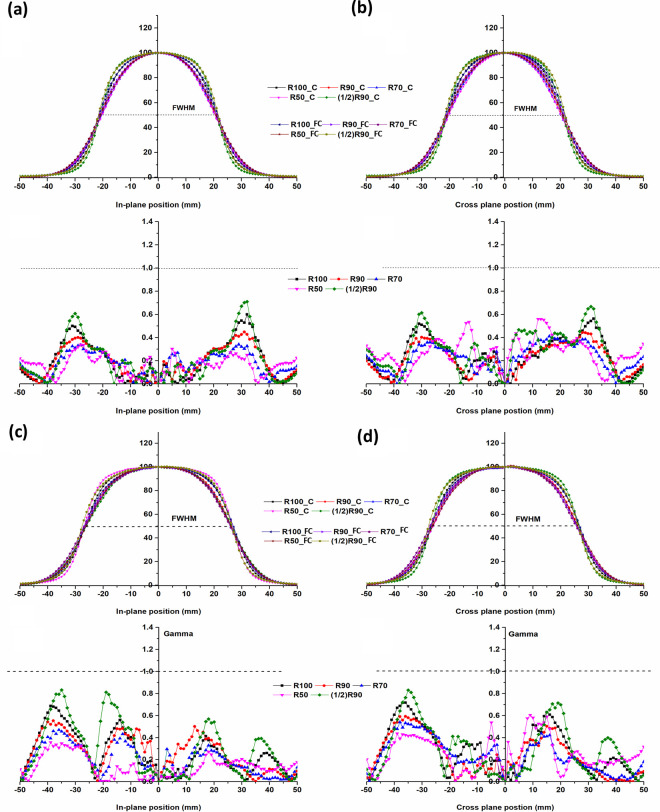
Plane-normalized profiles for 4, 5 cm diameter field and gamma index comparison (2%/ 2 mm) for applicators, at different depths. (a) In-plane of 4 cm, (b) Cross-plane of 4 cm, (c) In-plane of 5 cm, (d) Cross-plane of 5 cm (SSD: 100 cm. The dotted line represents a gamma index of 1.0 at 2%/2 mm).

Fig 6 and 7 show gamma evaluation (3%/3 mm) with differences between the beam profiles of the applicators measured at five measurement depths.

The difference in depth for all measurements between the applicators was within 1 mm (Table 2). The gamma evaluation (2%/2 mm) for consistency of the beam profile was passed depending on the depth.

In the tail region of the beam profiles, a slight difference was observed between the two applicators, particularly at shallow depths. This may be attributed to variations in beam scatter and collimation design between the fixed-size cone and the Cerrobend cutout-based applicator

### Output factor depending on field size for both applicators

Table 3 summarizes the OF measured in both applicators according to the field size and SSD. The OF (${OF}_{c}$) measured in the circular cerrobend applicator was higher than the OF (${OF}_{FC}$) measured in the fixed-size electron cone applicator for all field sizes regardless of the SSD. The average OF difference between the applicators was 2 cm (32.50 ± 6.73%), 3 cm (17.7 ± 6.56%), 4 cm (10.03 ± 3.78%), and 5 cm (7.88 ± 0.80%). Regardless of the SSD, it was confirmed that the OF difference between applicators decreased as the field size increased.

**Table 3:** Comparison of difference between applicators depending on field size (Diff. (%) * was calculated using equation 2).

	95 cm			100 cm			105 cm		
	${OF}_{c}$	${OF}_{FC}$	Diff. (%)*	${OF}_{c}$	${OF}_{FC}$	Diff. (%)*	${OF}_{c}$	${OF}_{FC}$	Diff. (%)*
2 cm	0.903	0.676	25.1	0.685	0.452	34.0	0.485	0.299	38.4
3 cm	0.970	0.861	11.2	0.790	0.650	17.7	0.632	0.478	24.4
4 cm	1.005	0.936	6.9	0.843	0.767	9.0	0.704	0.604	14.2
5 cm	1.012	0.939	7.2	0.867	0.791	7.7	0.742	0.644	8.8

### Comparison of dose distributions for applicators with gafchromic film

**Fig 8:**
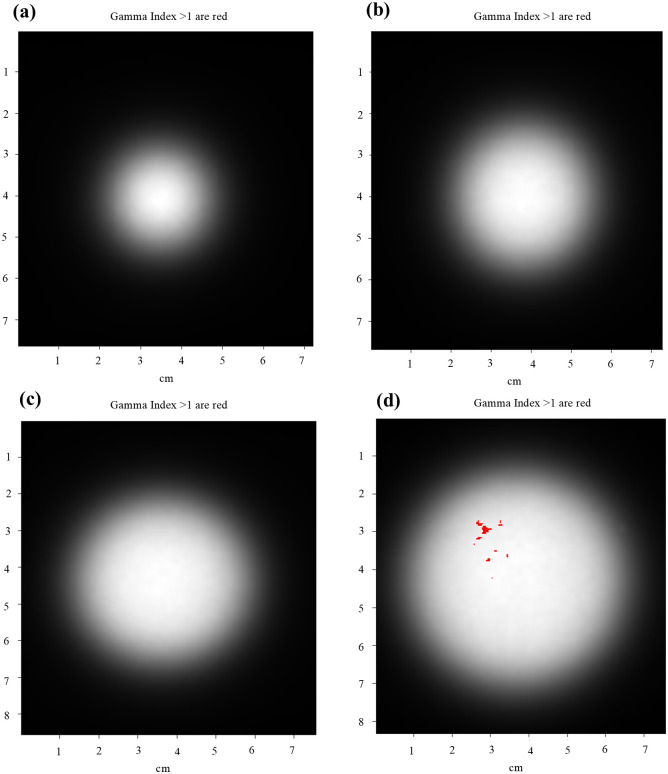
Gamma index comparison for 2D dose distributions of both applicators, obtained from film dosimetry software. Red indicates a gamma index > 1.0 (a) 2 cm, (b) 3 cm, (c) 4 cm, (d) 5 cm.

Fig 8 shows gamma evaluation (3%/3 mm) of the 2D dose distributions obtained for both applicators. In all field sizes, pass rates of 99% or higher were confirmed. Additional 2D dose distribution results obtained using Gafchromic film for both applicators are provided in S3 File (S1 Data)

### Comparison of monitor unit and dose distribution between applicator and TPS

Table 4 compares the MU calculated based on the measurements and MU calculated from TPS for both applicators. According to equation 3, MU (${MU}_{c},\;{MU}_{FC})$ was calculated using the PDD and OF measured in both applicators; ${MU}_{c\;from\;TPS}$ was calculated from the TPS in the same conditions as for the circular cerrobend-cutout applicator. Comparing ${MU}_{c\;from\;TPS}$with ${MU}_{c}$, it was confirmed that the differences were all within 2.0% (2 cm (1.67%), 3 cm (1.16%), 4 cm (0.55%), 5 cm (−0.36%)). ${MU}_{FC\;for\;TPS}$ was indirectly calculated using the fixed-size electron cone applicator for irradiating the same dose, using the ${MU}_{c\;from\;TPS}$ and the ${OF}_{relative}$ according to equation 4,5. To evaluate the reliability of ${MU}_{FC\;for\;TPS}$, the difference between ${MU}_{FC\;for\;TPS}$ and ${MU}_{FC}$ was compared; they were within 1.0% in a small electron field.

**Table 4:** Comparison of monitor units between two applicators and TPS (d: 14 mm, SSD: 100 cm).

$r_{a}$	type	OF (S_e_)	PDD (d, $r_{a}$, SSD,%)	MU	${OF}_{relative}$ $({OF}_{FC}$ / ${OF}_{c}$)	Diff. (%) of MU	
						${MU}_{c}\;$ vs ${MU}_{C\;from\;TPS\;}$	${MU}_{FC}\;$ vs ${MU}_{FC\;for\;TPS\;}$
2 cm	C	0.685	93.2	783	0.659	1.674	−0.228
	$C_{from\;TPS}$	–	–	770			
	FC	0.452	95.0	1165			
	${FC}_{for\;TPS}$	–	–	1168			
3 cm	C	0.790	99.1	638	0.822	1.159	0.708
	$C_{from\;TP}$			631			
	FC	0.650	99.5	773			
	${FC}_{for\;TPS}$	–	–	767			
4 cm	C	0.843	100.0	593	0.910	0.549	0.407
	$C_{from\;TPS}$			590			
	FC	0.767	100.0	651			
	${FC}_{for\;TPS}$	–	–	649			
5 cm	C	0.867	100.0	577	0.912	−0.357	−0.318
	$C_{from\;TPS}$			579			
	FC	0.791	100.0	632			
	${FC}_{for\;TPS}$	–	–	634			
10 cm × 10 cm ($r_{0})$	Ref.	1.000	1.000	500		–	–
	TPS	–	–	501		–	–

When the gamma evaluation (3%/3 mm) was performed in film dosimetry software, it was confirmed that there was a 99% or greater consistency between the 2D dose distribution obtained using film in the fixed-size electron cone applicator (${2D}_{FC})$ and the 2D dose distribution obtained from the TPS (${2D}_{c\;from\;TPS})$ (Table 5).

**Table 5:** Comparison of dose distributions between ${2D}_{FC}$ and ${2D}_{C\;rom\;TPS\;}$ using the gamma index.

	Pass rate of gamma evaluation (%)		
Field size	3%/ 3 mm	2%/2 mm	1%/1 mm
2 cm	100	100	73.16
3 cm	100	99.96	78.20
4 cm	100	99.34	79.18
5 cm	99.94	94.46	55.55

## Discussion

This study evaluated the possibility of dose calculation in the TPS for special applicators provided by equipment vendors for electron radiotherapy. This study provides a novel approach to dose calculation for non-standard electron applicators, potentially expanding TPS applicability in specialized treatment scenarios. Determining the MU of a special applicator provided by the vendor for special treatment requires considerable time, and cannot be directly performed in the TPS. To overcome these disadvantages, this study proposes a method of calculating the dose distribution and MU for special applicators in the TPS using an indirect method analyzing the difference in the dose characteristics between the applicators.

The concept of this study is based on the results of a study by Venanzio et al [6]. Their study evaluated the efficiency of a newly developed single-crystal diamond diode (SCDD) for reliable dosimetry of small electron fields. The dosimetric characteristics were evaluated for both applicators for electron radiotherapy. Their study confirmed that there was a similarity in dosimetric characteristics between the applicators but did not present the difference quantitatively. The detectors (microdiamond detectors) used in this study were market versions of the SCDD and were used for all measurements. Microdiamond detectors have been used by many researchers to verify electron beams [3,10–12].

Unlike previous dosimetric studies, such as that by Venanzio et al., the present work focuses on the integration of fixed-size electron cone applicators into a commercial TPS for routine clinical application. This includes practical methods for MU calculation, dose visualization, and treatment planning using CT-based simulations, which have not been previously demonstrated.“

And this study was conducted using only a 6 MeV beam. Gamma evaluation was performed using a gafchromic film to verify the difference in the 2D dose distributions between the two applicators.

It was confirmed that gamma evaluations for beam profile consistency using microdiamond detectors met the 2%/2 mm criterion for 100% of points. For 2D distributions with gafchromic film, the 3%/3 mm criterion was met for more than 99% of points. There was no difference in dose distribution between the applicators; however, there was a significant difference in the OF (Tables 3). The OF difference between the two applicators was attributed to their structural design and material composition. This difference may have clinical significance, as variations in OF could impact dose delivery accuracy in treatment planning. Venanzio et al. [6] only compared the OF difference in the reference SSD (100 cm); in this study, it was evaluated depending on the SSD. The OF difference between the applicators was confirmed to decrease as the field size increased, regardless of the SSD. A detailed statistical analysis of this trend could further validate the robustness of the findings. OF was defined using the value measured at depth $R_{100}$ previously determined from the PDD (Equation 1). When the PDD consistency between the applicators was evaluated, there was a high consistency of 1%/1 mm except for the surface; the difference was within 1 mm, regardless of the type of applicator (Table 1).

The relative OF presented in this study was proposed by Rusk et al. [13] as the output correction factor (OCF). They suggested a copper cutout to replace the cerrobend cutout used in electron radiotherapy, and evaluated the dosimetric characteristics of the two materials. The OCF was used to deliver the same dose to the copper material cutout. When they compared the OF according to the cutout material in the same applicator, it was found that there was a difference in energy, within 1% at 6 MeV. Gamma evaluation (1%/2 mm) was used to analyze the differences in dose distribution. Similar to the concept of this study, Rusk et al. [13] confirmed the possibility of dose calculation without considering the difference in material in the cutout in the TPS through quantitative analysis of the differences in dosimetric characteristics between the two materials. Although they suggested the possibility of dose calculation using a copper cutout in the TPS, an additional study was not performed. However, this study evaluated the possibility of dose calculation for a fixed-size electron cone applicator in a TPS. This possibility was confirmed through comparative analysis of the MU and dose distribution for the applicator and the TPS (Tables 4 and 5).

The algorithms used in the TPS are generally the Hogstrom pencil beam and eMC-based Monte Carlo for electron radiotherapy. The study’s use of the eMC algorithm enhances calculation accuracy, yet further validation with other algorithms is recommended. From previous studies [14,15], the pencil beam algorithm does not recommend MU calculation in a small electron field, and the MU calculation is not accurate in electron radiotherapy containing high density materials [16,17]. Considering the uncertainty of dose calculations, such as with the pencil beam algorithm in a small electron field, the MU in this study was calculated using the MC-based eMC algorithm.

In this study, compared with the MU calculated based on the measurements using the eMC algorithm of the TPS, it was confirmed that there was a difference of less than 2.0% in small electron fields. However, it can be confirmed that there was a difference from the results in the study by Richmond et al. [18] and that there was a difference of less than 2.0% in small fields of 3 cm or more. Huang et al. [19] and Chamberland et al. [20] were similar to those in this study.

Due to the structural constraints of the RayStation eMC algorithm, manual entry of output or cutout factors for custom applicators is not supported. All beam parameters, including output factors, are derived automatically based on measured PDD and profile data during commissioning [21]. Therefore, direct modeling of the fixed-size electron cone applicator as an independent entity within the TPS is not feasible in this study.

Considering the limitations of the RayStation TPS in independently modeling fixed-size electron cone applicators, a scaling factor approach was used to accurately reflect the measured output differences. This method preserves the integrity of the validated TPS beam model while enabling accurate MU calculation for fixed-size electron cone applicators in clinical settings

For electron radiotherapy, a general applicator-based cerrobend cutout provided by a vendor can be used to determine the MU using a commercial TPS. A limitation of this study is the use of a single energy level (6 MeV). Future studies could explore the impact of energy variations and different electron applicator materials to further validate the findings. A special applicator that cannot be used in the TPS despite advantages in clinical practice requires considerable time to determine the MU, making it difficult to use in clinical practice.

This study demonstrated a clinically feasible method to incorporate fixed-size electron cones into a commercial TPS (RayStation) for small-field electron beam therapy. Our approach enables monitor unit (MU) calculation and dose visualization without the need for Cerrobend cutouts, thereby offering a simplified and efficient workflow for clinical use.

Although out-of-field dose analysis was not included in this study, measurements have already been performed and will be analyzed in a follow-up publication. This future work will incorporate existing findings on non-zero peripheral dose in electron treatments [22], and evaluate second cancer risks using established dose-risk models such as ICRP Publication 103 [23].

## Conclusion

This study was conducted to evaluate the feasibility of using a treatment planning system (TPS) to determine monitor units (MUs) for specialized electron applicators, improving the efficiency of MU calculations in clinical practice. By leveraging the MU from a standard applicator in the TPS, we estimated the MU for a non-standard applicator using an indirect calculation approach. The dosimetric differences between applicators remained within clinically acceptable limits, ensuring accuracy and reliability in MU determination for small electron fields. These findings confirm the potential of using TPS-based dose calculations for specialized electron beam applicators, reducing manual workload and enhancing clinical workflow efficiency.

## Supporting information

S1 Data **S1 File.** Supplementary slides summarizing the design and dosimetric characteristics of Cerrobend and fixed-size electron applicators. **S2 Table.** Raw measurement data including output factors and depth dose values for various field sizes at 6 MeV. **S3 File.** 2D dose distribution images acquired using Gafchromic film for both applicator types under various field sizes.
(ZIP)
