# Supplementary material for: Feasibility study for dose calculation with a radiation treatment planning system using a fixed-size electron cone applicator for small electron fields
Source: PLoS One. 2025 Aug 14;20(8):e0324722. doi: 10.1371/journal.pone.0324722 (PMC12352773; doi:10.1371/journal.pone.0324722)
Supplement: S1 Data — S1 File. Supplementary slides summarizing the design and dosimetric characteristics of Cerrobend and fixed-size electron applicators. S2 Table. Raw measurement data including output factors and depth dose values for various field sizes at 6 MeV. S3 File. 2D dose distribution images acquired using Gafchromic film for both applicator types under various field sizes. (ZIP) [file pone.0324722.s001.zip › Support data_ Revised/S1_Both applicators for electron radiotherapy.pptx]

## Slide 1
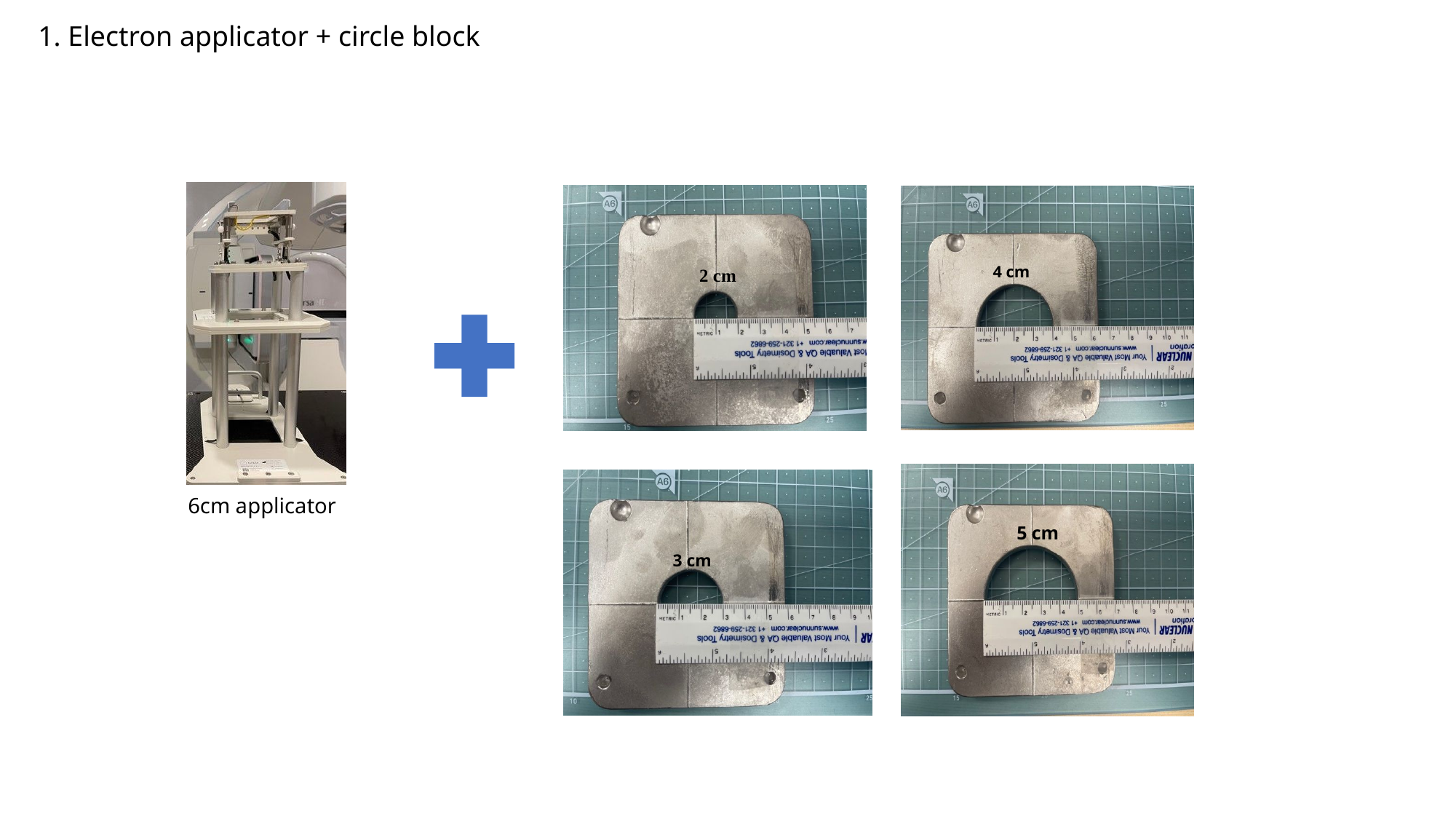

1. Electron applicator + circle block
4 cm
2 cm
5 cm
3 cm
6cm applicator

## Slide 2
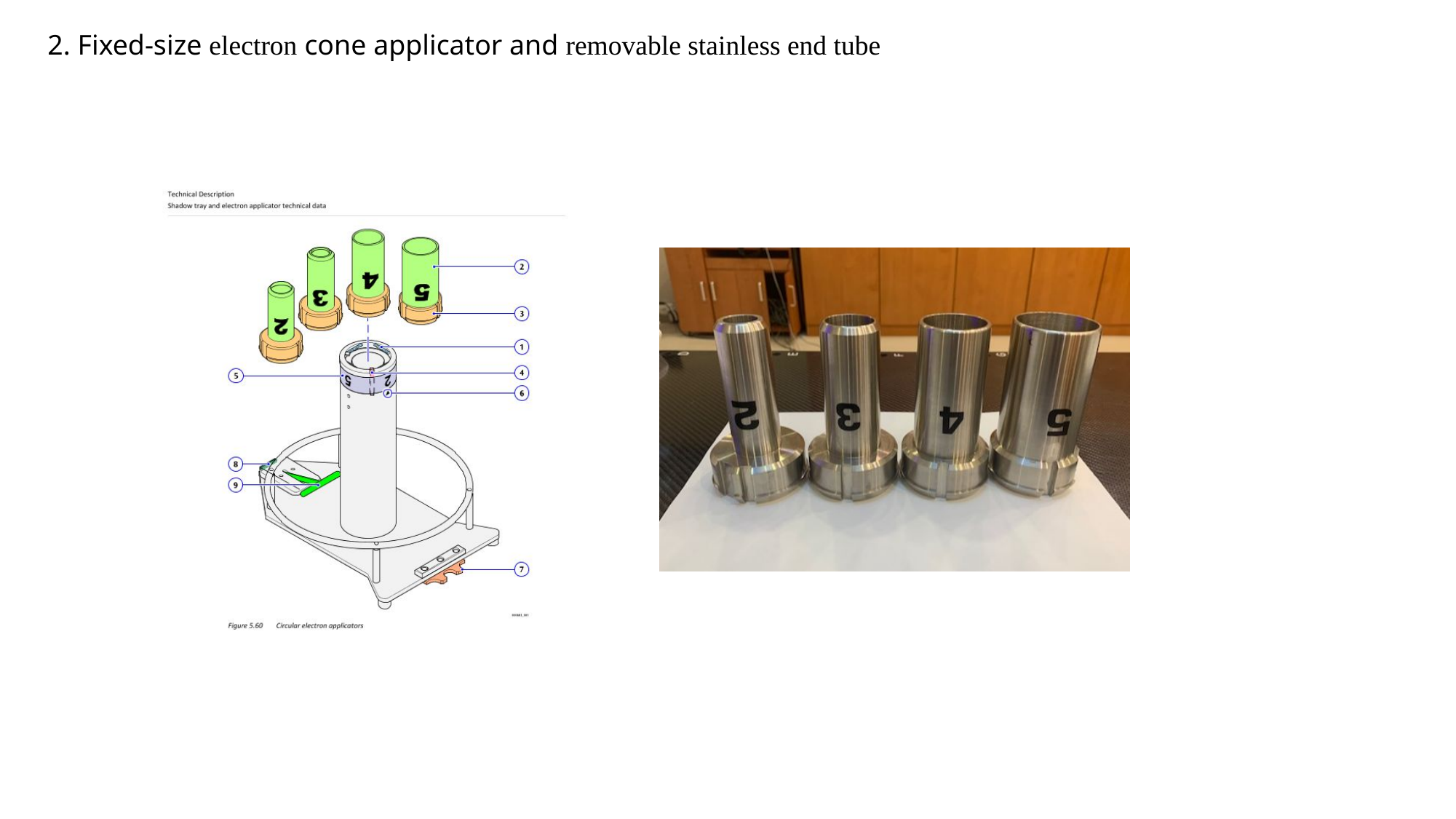

2. Fixed-size electron cone applicator and removable stainless end tube
