# Supplementary material for: Feasibility study for dose calculation with a radiation treatment planning system using a fixed-size electron cone applicator for small electron fields
Source: PLoS One. 2025 Aug 14;20(8):e0324722. doi: 10.1371/journal.pone.0324722 (PMC12352773; doi:10.1371/journal.pone.0324722)
Supplement: S1 Data — S1 File. Supplementary slides summarizing the design and dosimetric characteristics of Cerrobend and fixed-size electron applicators. S2 Table. Raw measurement data including output factors and depth dose values for various field sizes at 6 MeV. S3 File. 2D dose distribution images acquired using Gafchromic film for both applicator types under various field sizes. (ZIP) [file pone.0324722.s001.zip › Support data_ Revised/S3_Both applicators 2D dose distribution with gafchromic film.pptx]

## Slide 1
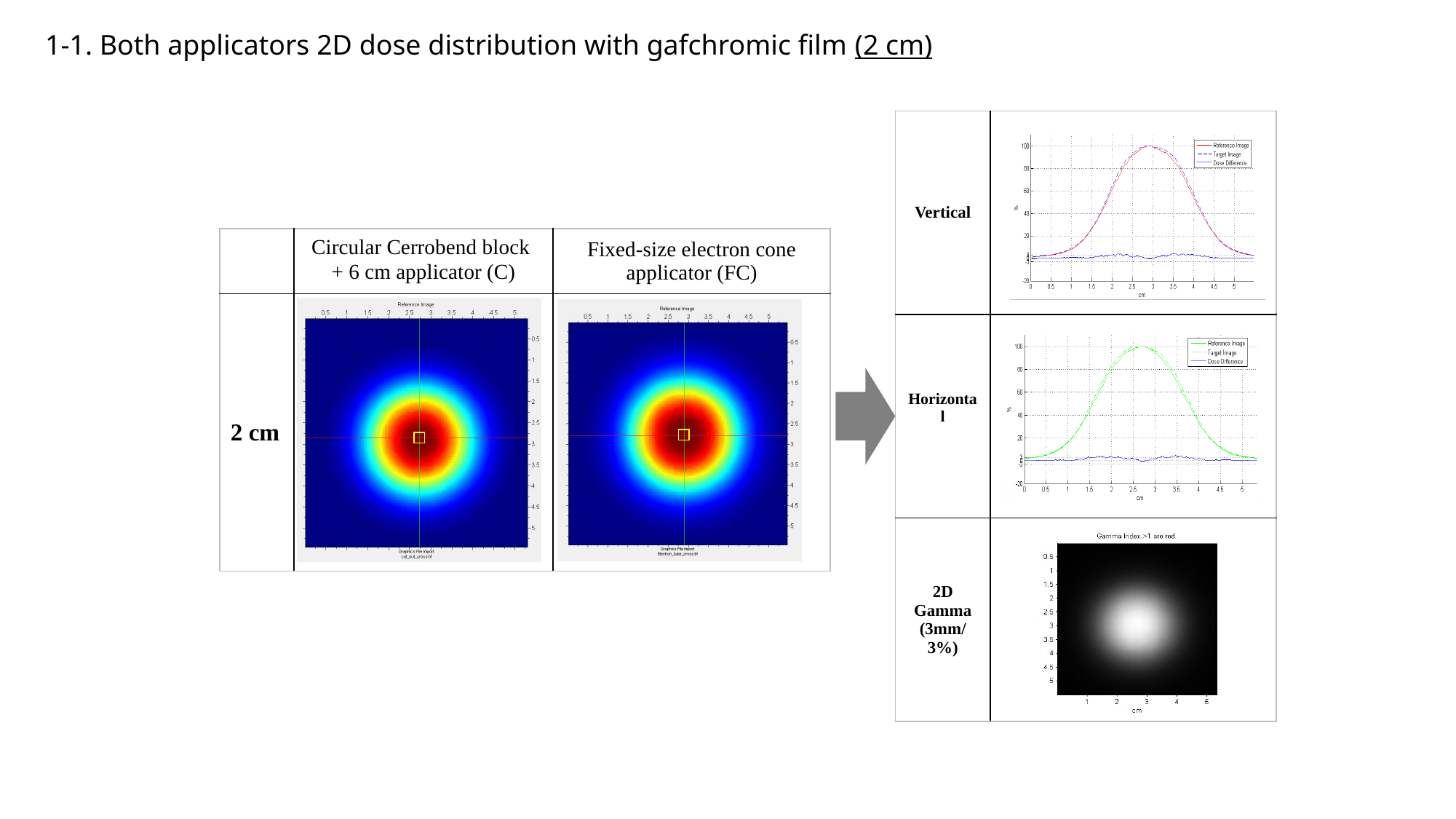

1-1. Both applicators 2D dose distribution with gafchromic film (2 cm)
| Vertical | |
| --- | --- |
| Horizontal | |
| 2D Gamma (3mm/3%) | |
| | Circular Cerrobend block + 6 cm applicator (C) | Fixed-size electron cone applicator (FC) |
| --- | --- | --- |
| 2 cm | | |
100.6±0.51%
98.2±0.27%

## Slide 2
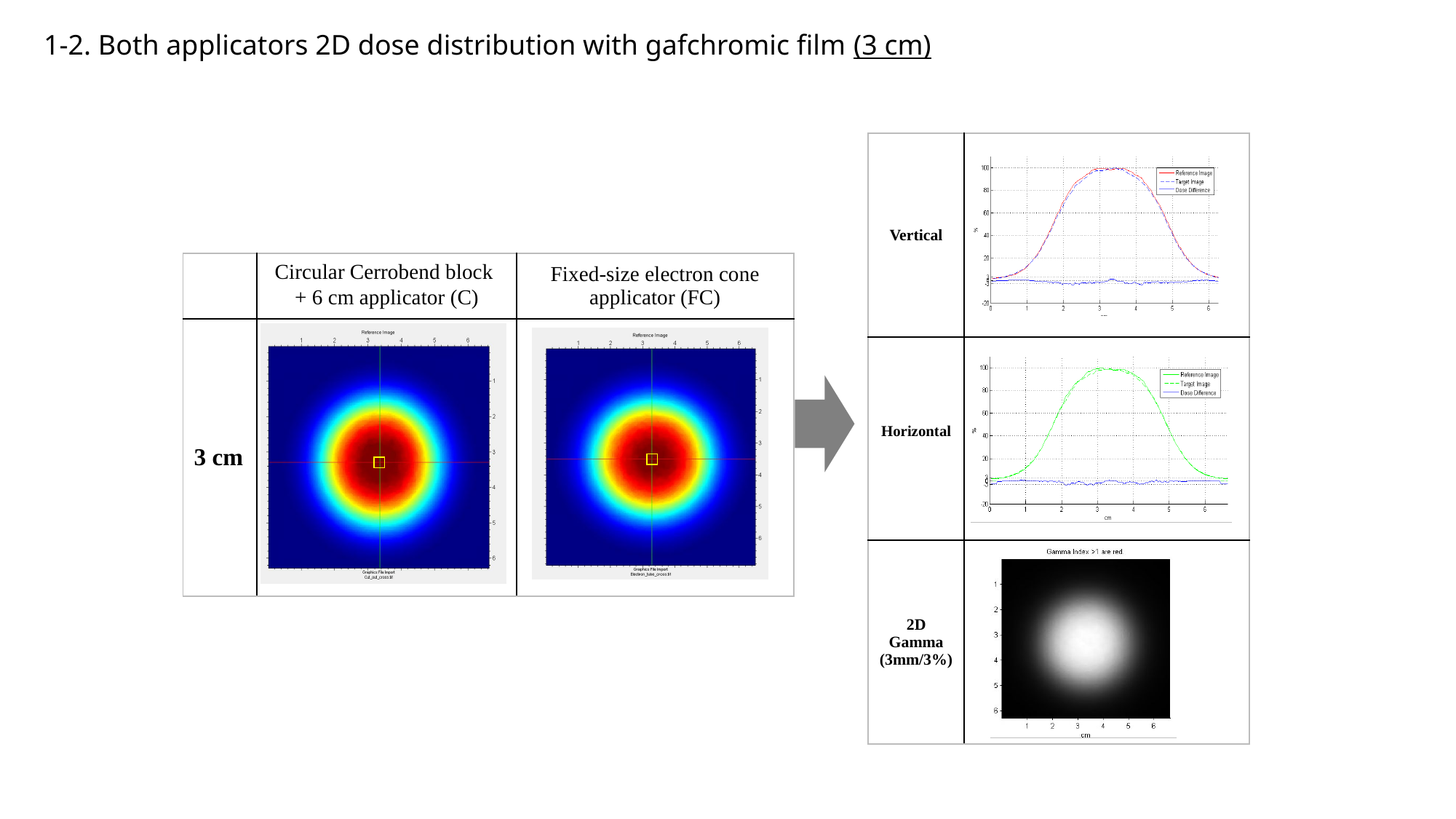

1-2. Both applicators 2D dose distribution with gafchromic film (3 cm)
| Vertical | |
| --- | --- |
| Horizontal | |
| 2D Gamma (3mm/3%) | |
| | Circular Cerrobend block + 6 cm applicator (C) | Fixed-size electron cone applicator (FC) |
| --- | --- | --- |
| 3 cm | | |
98.8±0.30%
99.6±0.32%
101.5%

## Slide 3
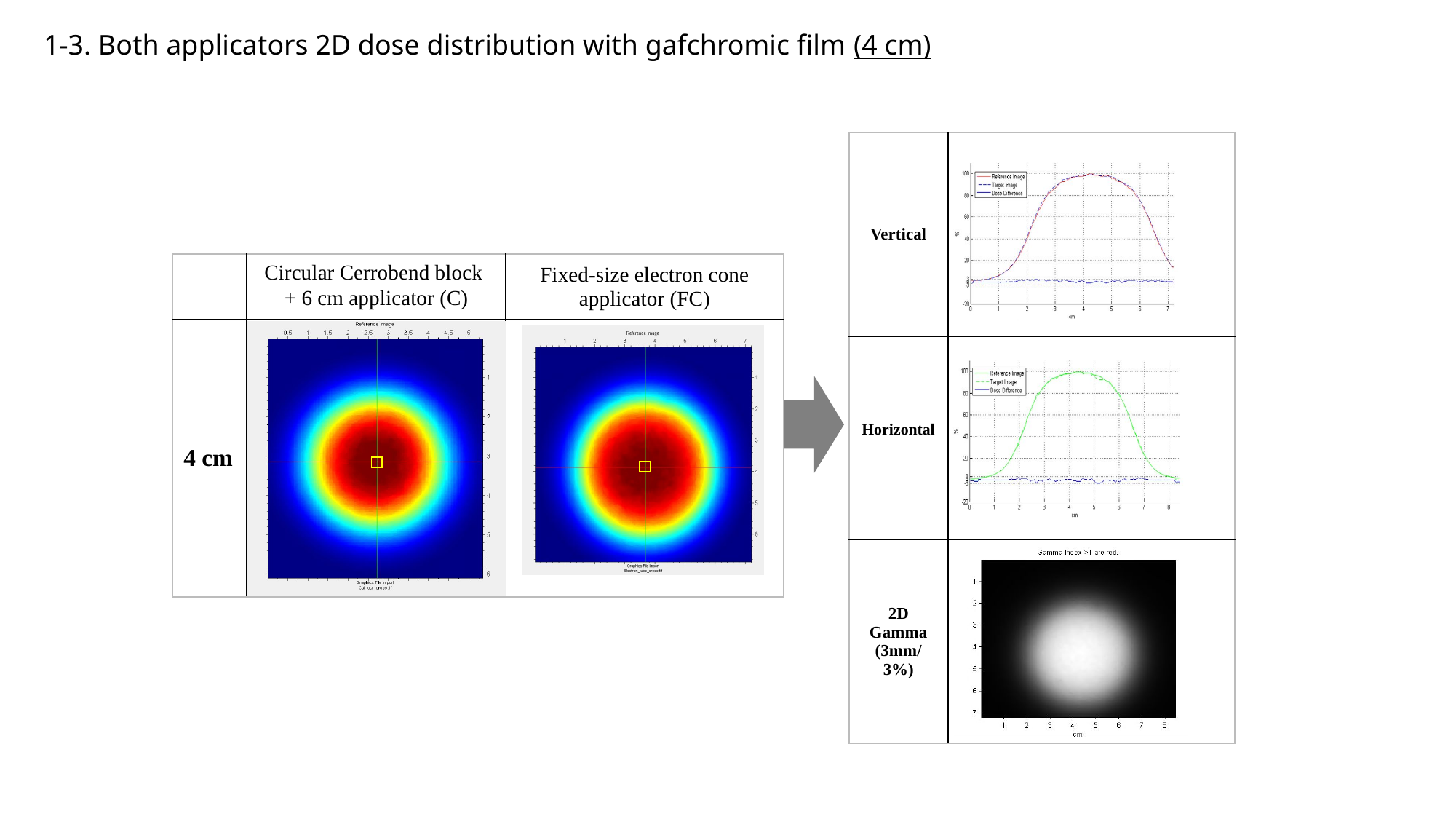

1-3. Both applicators 2D dose distribution with gafchromic film (4 cm)
| Vertical | |
| --- | --- |
| Horizontal | |
| 2D Gamma (3mm/3%) | |
| | Circular Cerrobend block + 6 cm applicator (C) | Fixed-size electron cone applicator (FC) |
| --- | --- | --- |
| 4 cm | | |
99.2±0.46%
101.3±0.23%
101.5%

## Slide 4
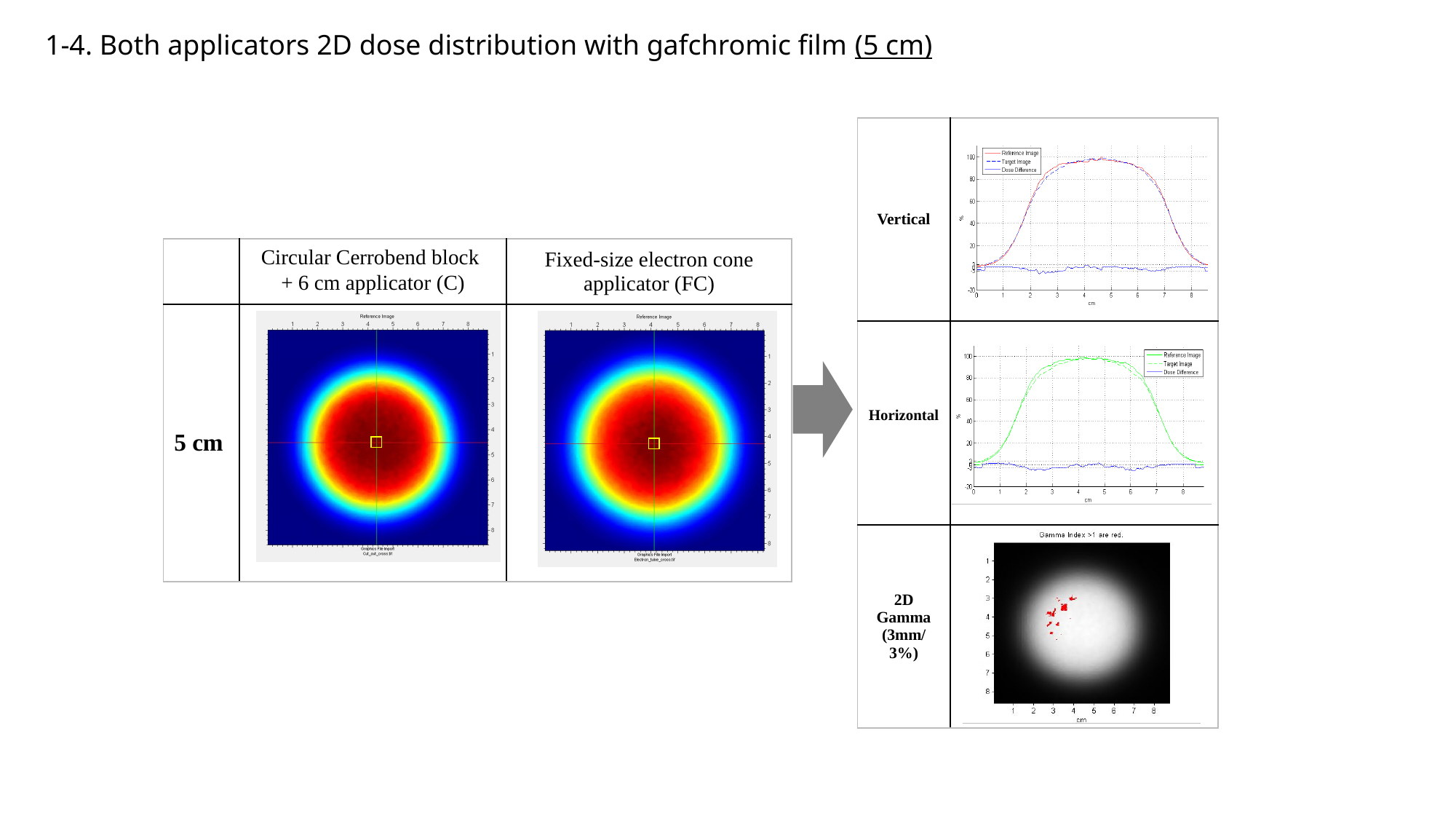

1-4. Both applicators 2D dose distribution with gafchromic film (5 cm)
| Vertical | |
| --- | --- |
| Horizontal | |
| 2D Gamma (3mm/3%) | |
| | Circular Cerrobend block + 6 cm applicator (C) | Fixed-size electron cone applicator (FC) |
| --- | --- | --- |
| 5 cm | | |
99.7±0.51%
100.6±0.48%
101.5%
